# Alterations of circulating lymphocyte subsets in patients with colorectal carcinoma

**DOI:** 10.1007/s00262-021-03127-8

**Published:** 2021-12-20

**Authors:** Johanna Waidhauser, Pia Nerlinger, Tim Tobias Arndt, Stefan Schiele, Florian Sommer, Sebastian Wolf, Phillip Löhr, Stefan Eser, Gernot Müller, Rainer Claus, Bruno Märkl, Andreas Rank

**Affiliations:** 1 Department of Hematology and Oncology, University Medical Center Augsburg, Stenglinstr.2, 86156 Augsburg, Germany; 2 Faculty of Mathematics and Natural Sciences, Institute of Mathematics, University of Augsburg, Augsburg, Germany; 3 Department of General, Visceral and Transplant Surgery, University Medical Center Augsburg, Augsburg, Germany; 4 Department of Gastroenterology, University Medical Center Augsburg, Augsburg, Germany; 5 General Pathology and Molecular Diagnostics, Medical Faculty, University of Augsburg, Augsburg, Germany

**Keywords:** Circulating lymphocytes, Cellular immune status, Colorectal carcinoma, Tumor immune response, Flow cytometry

## Abstract

**Introduction:**

Cellular immune response to cancer is known to be of great importance for tumor control. Moreover, solid tumors influence circulating lymphocytes, which has been shown for several types of cancer. In our prospective study we elucidate changes in lymphocyte subsets in patients with colorectal carcinoma compared to healthy volunteers.

**Methods:**

Flow cytometry was performed at diagnosis of colon carcinoma to analyze B cells, T cells and NK cells including various subtypes of each group. Univariate and multivariate analyses including age, gender, tumor stage, sidedness and microsatellite instability status (MSI) were performed.

**Results:**

Forty-seven patients and 50 healthy volunteers were included. Median age was 65 years in patients and 43 years in the control group. Univariate analysis revealed lower total lymphocyte counts, lower CD4 + cells, CD8 + cells, B cells and NKs including various of their subsets in patients. In multivariate analysis patients had inferior values of B cells, CD4 + cells and NK cells and various subsets, regardless of age and gender. Naïve, central memory and HLADR + CD8 + cells showed an increase in patients whereas all other altered subsets declined. MSI status had no influence on circulating lymphocytes except for higher effector memory CD8 + cells in MSI-high patients. Localization in the left hemicolon led to higher values of total cytotoxic T cells and various T cell subsets.

**Conclusion:**

We found significant changes in circulating lymphocyte subsets in colon carcinoma patients, independent of physiological alterations due to gender or age. For some lymphocyte subsets significant differences according to tumor localization or MSI-status could be seen.

**Supplementary Information:**

The online version contains supplementary material available at 10.1007/s00262-021-03127-8.

## Introduction

The impact of cellular immune response on local and systemic tumor control has been of great interest not only since the era of checkpoint inhibition for tumor therapy. The tumor microenvironment consisting of immune cells and non-immune cells such as endothelial and stromal cells has a non-negligible impact on the prognosis of cancer patients [1]. However, not only local infiltration of immune cells is altered by the presence of tumor cells. Circulation of immune cells, especially the composition of lymphocyte subsets was shown to underly significant changes in patients with cancer. In patients with non-small-cell lung cancer (NSCLC), CD3 + and CD4 + lymphocytes in peripheral blood decreased significantly compared to healthy controls [2]. The presence of metastatic inflammatory breast cancer [3] as well as hepatocellular carcinoma [4] was associated with significant reduction of T- and B cells. For patients with colorectal carcinoma few studies exist regarding alterations of lymphocytes and their subsets compared to healthy controls and most of them focus on circulating immune factors as cytotoxic T lymphocyte antigen 4 (CTLA-4) or expression levels of certain cytotoxicity receptors on NK cells and not on lymphocyte subsets [5, 6]. Only Spacek et al. focused on peripheral blood lymphocytes and detected significant declines of main lymphocyte subgroups in CRC patients compared to healthy controls [7]. Nevertheless, not only cancer itself affects the composition of peripheral blood cellular immune status, but also age and gender are known to influence lymphocyte subsets [8–10]. In colorectal carcinoma particularly, another relevant factor with regard to the immune response to tumors is the microsatellite status, a surrogate for mismatch repair deficiency (MMRd), which is divided into microsatellite status with high instability (MSI-H) and microsatellite stable (MSS) [11]. MSI-H tumors are considered to carry increased tumor mutational burden (TMB) and to be of increased immunogenicity, thus better detectable for the individual immune system [12, 13]. However, detailed analyses of the influence of MSI status on circulating lymphocytes do not exist. Additionally, the localization of the tumor in the right or left hemicolon harbors different molecular and phenotypic characteristics including differences in immunogenicity [14, 15]. The influence of these factors has hardly been considered when regarding the cellular immune system of cancer patients. The aim of our study was to elucidate changes in total peripheral lymphocyte counts, B cells, T cells and NK cells with various subsets as well as the impact of age, gender, MSI status and tumor sidedness in patients with localized and metastasized colorectal cancer compared to healthy individuals.

## Patients and methods

### Study population and trial design

Patients who were diagnosed with local or locally advanced colorectal carcinoma and scheduled for surgery at University Medical Center Augsburg between December 2018 and November 2020 were included. Exclusion criteria were a history of chronic infectious disease, inherent or acquired immunodeficiency or autoimmune disorder and immunosuppressive therapy. Patients who turned out to have a metastatic disease during or after surgery were included in the analysis as long as the tumor was resected. Additionally a control group of 50 healthy volunteers, mostly blood donors from the blood bank of University Medical Center Augsburg were recruited. According to the national guidelines of blood donation healthy controls underwent a routine anamnesis regarding preexisting medical conditions. Therefore the likelihood of chronic diseases or the existence of an undetected colorectal carcinoma in the control group is low.

The study was approved by the medical ethical committee of Ludwigs Maximilians University Munich (reference number 18–726) and written informed consent was obtained from all patients and healthy participants.

### Analysis of lymphocytes and subsets

Blood samples (EDTA blood) were taken preoperatively at the time of first surgical presentation to perform flow cytometry (FC500 from Beckman Coulter, Brea, California, USA) in our local laboratory within 24 h. Cell staining was done using commercial fluoreszeinisothiocyanat (FITC-), phycoerythrin (PE-), phycoerythrin Texas red-X (ECD-) and phycoerythrin-cyanin (PC5- and PC7-) labeled antibodies purchased from Beckman Coulter (Brea, California, USA) and Biolegend (San Diego, California, USA). Initial results for lymphocyte subsets were given as percentages. Absolute values were calculated using absolute leucocyte counts measured with Stem-Count (Stem-Kit, Beckman Coulter). Analysis of the different lymphocyte subsets was done as previously reported by our research group [16–18].

B lymphocytes were identified by the presence of CD19 (CD19-PC7 IM3628) and were further divided into naïve (IgD + CD27-; IgD-FITC B30652, CD27-ECD B26603), memory (IgD + CD27 +), class switched memory (IgD- CD27 +) and transitional (CD24hi CD38hi; CD24-PE IM1428U, CD38-PC5 A07780) subsets.

T lymphocytes defined by positivity for CD8 or CD4 were subdivided into naïve (CD62L + CD45RA +), memory T cells (CD4 + CD45RA- CD45RO + /CD8 + CD45RA- CD45RO +), which were further divided into central memory (CD62L + CD45RA-), effector memory (CD62L- CD45RA-), effector memory RA + (EMRA) (CD62L- CD45RA +) and activated memory (HLA-DR + or CD69 +) cells, and regulatory (FoxP3 +) and IL2R + cells.

Furthermore, type 1, 2 and 17 CD4 + T helper (Th1/Th2/Th17) cells were identified by using antibodies against CXCR3, CCR4, CCR5 and CCR6. Th1 cells were defined as CD4 + CXCR3 + CCR4- CCR5 + CCR6-, Th2 cells as CD4 + CXCR3- CCR4 + CCR5- CCR6- and Th17 cells as CD4 + CXCR3- CCR4 + CCR5- CCR6 + .

Within cytotoxic CD8 + T lymphocytes, activated subsets in early (CD28 + CD27 +), intermediate (CD28- CD27 +) and late (CD28- CD27-) status were identified as well as exhausted (CD279 +) and terminal effector (CD279- CD57 +) cells. Additionally, CD56 + CD3 + T cells were registered.

NK lymphocytes were detected as CD56 + cells and subdivided into 3 NK subsets (CD56 + CD16 + , CD56dim CD16bright, and CD56bright CD16dim).

Detailed information regarding antibodies and gating strategy is displayed in the supplement Figure S1 [18] and Table S1.

### Microsatellite status

Immunohistochemistry with expression analysis of PMS2 and MSH6 was used to detect microsatellite instability in the resected tumors.

### Statistical analysis

The results of descriptive analysis are given as median values and interquartile ranges. To detect differences between patients and the control group as well as female and male study population univariate analysis using Mann–Whitney-U test was performed. Age-dependent alterations of lymphocyte subsets were analyzed after logarithmic transformation. Chi-square test was used to compare frequencies of MSI-H and MSS patients in right and left sided colorectal carcinomas. Additionally, a multivariate linear regression analysis was performed on log-transformed cell counts including tumor stage, age and gender and in a second step for patients only including age, gender, tumor stage, microsatellite instability (MSI) and sidedness of the tumor. The results of multivariate analysis were given as multiplicative factors (coefficient B). *p* values < 0.05 were considered statistically significant. Data were analyzed with SPSS for Windows (IBM SPSS Statistics 24, Armonk, New York, USA) and R 4.0.2.

## Results

### Population characteristics

A total of 47 patients with colorectal cancer and 50 healthy volunteers were included. In the patient group median age was 65 years (range: 42–84) and 18 patients (38%) were female. Participants of the control group were younger with a median age of 43 years (range: 18–81; *p* < 0.000), 17 (34%) were women. The significant age difference was also addressed by including this factor into the multivariate analysis (see below).

Patients in all four UICC stadiums were included (UICC I: *n* = 11; UICC II: *N* = 18; UICC III: *n* = 13; UICC IV: *n* = 5). All patients in UICC stadium IV underwent surgery unaware of the metastatic situation. The localization of the tumor was in the right hemicolon (coecum, colon ascendens and colon transversum up to splenic flexure) in 32 patients and in the left hemicolon (colon descendens and sigma) [14, 19–21] in 15 patients. 11 patients presented with microsatellite instability. Table 1

**Table 1:** Demographic and disease characteristics of patients and control group

Variables	Patients (*n* = 47)	Control group (*n* = 50)	*P* value
Age; *median (range)*	65 (42–84)	43 (18–81)	0.00
*Gender*			
Male; *n* (%)	29 (62)	33 (66)	0.661
Female; *n* (%)	18 (38)	17 (34)	
*Stage*			
UICC I; *n* (%)	11 (23)		
UICC II; *n* (%)	18 (38)		
UICC III; *n* (%)	13 (28)		
UICC IV; *n* (%)	5 (11)		
*Tumor sidedness*			
Right *n* (%)	32 (68)		
Left *n* (%)	15 (32)		
*Microsatellite status*			
Stable *n* (%)	35 (74)		
Instable *n* (%)	11 (23)		

## Univariate analysis

**Fig. 1:**
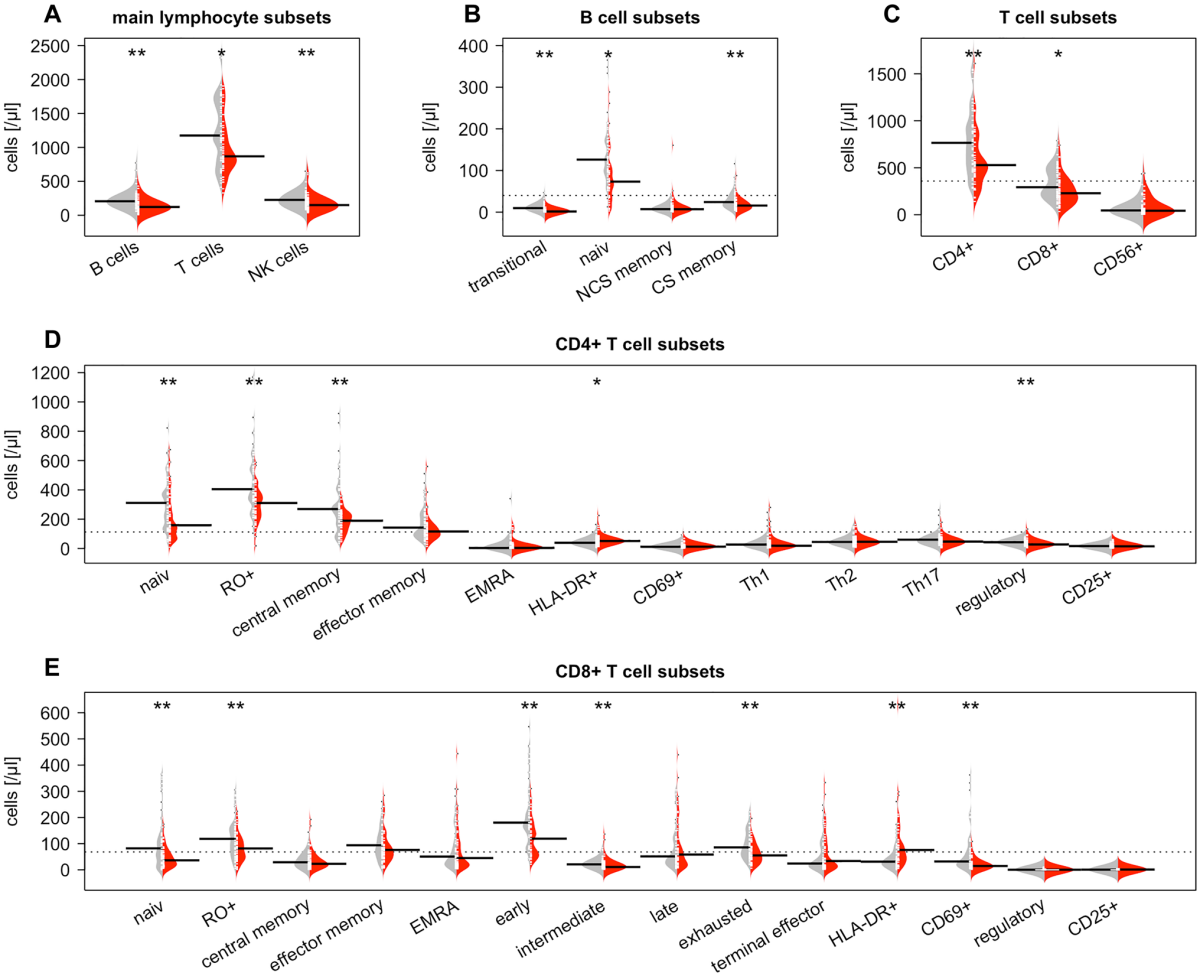
Comparison of lymphocyte subsets between colon carcinoma patients (red) and healthy controls (gray). **A** Main lymphocyte subsets, **B** B cell subsets, **C** T cell subsets, **D** CD4 + T cell subsets, **E** CD8 + T cell subsets. White ticks indicate the individual data points; dotted lines show the overall subset average. Significant *p* values are displayed with * for *p* < 0.05 and ** for *p* < 0.005

Univariate analysis revealed significant differences with lower values for total lymphocyte count in carcinoma patients compared to healthy controls (median: 1320/µl vs. 1878/µl; *p* = 0.000). CD8 + T cells were decreased in tumor patients (229/µl vs. 292/µl; *p* = 0.009) as well as CD4 + T cells (528/µl vs. 765/µl; *p* = 0.001). Regarding T cell subsets significantly lower values were observed in the patient group for naïve and memory CD8 + cells and early, intermediate and exhausted cytotoxic T cells and for naïve, memory, central memory and regulatory CD4 + cells. HLA-DR positive CD4 + and CD8 + showed significantly higher values in tumor patients. Total NK cell counts were reduced in colorectal cancer patients (122/µl vs. 226/µl; *p* = 0.002) as well as NK cell subsets (CD56 + CD16 + : 129/µl vs. 193/µl; *p* = 0.002; CD56dim CD16bright: 9/µl vs. 15/µl; *p* = 0.42; CD56bright CD16dim: 11/µl vs. 15/µl; *p* = 0.002). Cancer patients also had lower numbers of B lymphocytes (122/µl vs. 206/µl; *p* = 0.000) and lower values for most of the B cell subsets Fig. 1, Table 2.

**Table 2:** Lymphocyte subsets in healthy controls and patients. Cell counts are given as median value/ µl (interquartile range). P values are given for univariate analysis between total group of cancer patients and healthy controls

	Healthy controls Median cell count (interquartile range) (*n* = 50)	Colon carcinoma patients Median cell count (interquartile range) (*n* = 47)	*p* value univariate analysis
*Total lymphocytes*	1878 (1282−2292)	1320 (1046−1666)	**0.000**
CD3 + cells	1175 (806−1678)	868 (714−1190)	**0.011**
CD8 + cells	292 (203−493)	229 (131−344)	**0.009**
Naive	82 (35−140)	36 (16−67)	**0.004**
Memory	118 (63−165)	82 (45−118)	**0.008**
CM	29 (13−55)	23 (13−50)	0.563
EM	94 (55−143)	76 (45−112)	0.196
EMRA	51 (22−128)	45 (17−114)	0.623
Early	180 (130−295)	119 (58−169)	**0.000**
Intermediate	21 (12−34)	11 (6−26)	**0.006**
Late	51 (26−124)	58 (19−150)	0.931
Exhausted	86 (54−136)	54 (29−89)	**0.002**
Terminal effector	24 (12−95)	34 (11−117)	0.697
Regulatory	0 (0−1)	0 (0−1)	0.277
IL2	1 (0−1)	1 (0−2)	0.328
HLA-DR	31 (17−61)	76 (37−141)	**0.000**
CD69	32 (17−89)	15 (8−25)	**0.000**
CD4 + cells	765 (525−1017)	528 (400−768)	**0.001**
Naive	311 (173−416)	159 (79−295)	**0.002**
Memory	404 (309−590)	311 (214−378)	**0.000**
CM	269 (157−402)	190 (124−238)	**0.003**
EM	143 (93−238)	116 (78−167)	0.097
EMRA	4 (1−32)	5 (2−19)	0.636
Th1	27 (14−58)	19 (10−40)	0.156
Th2	44 (30−67)	46 (32−62)	0.917
Th17	60 (35−82)	47 (33−67)	0.081
Regulatory	43 (29−60)	28 (19−43)	**0.002**
Il2R +	17 (8−21)	15 (8−25)	0.874
HLA-DR	39 (27−60)	52 (44−65)	**0.015**
CD69	12 (7−19)	13 (9−21)	0.274
CD3 + CD56 + cells	45 (18−81)	43 (19−108)	0.945
*NK cells*	226 (139−300)	150 (87−223)	**0.002**
CD56 + CD16 +	193 (111−274)	129 (65−203)	**0.002**
CD56dim CD16bright	15 (11−20)	9 (7−18)	**0.042**
CD56bright CD16dim	15 (10−19)	11 (8−14)	**0.002**
*B cells*	206 (146−276)	122 (69−185)	**0.000**
Naive	126 (90−172)	73 (37−113)	**0.000**
Non-class-switched Memory	7 (5−13)	7 (3−14)	0.328
Class switched	24 (14−43)	16 (8−24)	**0.001**
Transitory	10 (5−18)	2 (1−4)	**0.000**
CD4/CD8 Ratio	2.4 (1.6–3.3)	2.2 (1.8−3.3)	0.997

Bold values indicate lymphocyte subsets with significant differences (*p* < 0.005)

Dividing the cancer patients into two subgroups (localized disease: UICC stadium I and II, and advanced disease: UICC stadium III and IV) revealed differences between localized tumor group and the healthy controls for HLA-DR positive CD4 + cells (55/µl vs. 39/µl; *p* = 0.003), total CD8 + cells (224/µl vs. 291/µl; *p* = 0.010), memory CD8 + cells (69/µl vs. 118/µl; *p* = 0.004), effector memory CD8 + cells (67/µl vs. 93/µl; *p* = 0.044) and exhausted CD8 + cells (55/µl vs. 86/µl; *p* = 0.002), as well as NK cells (147/µl vs. 226/µl; *p* = 0.003) and their subsets of CD56dimCD16bright (9/µl vs. 15/µl; *p* = 0.028) and CD56brightCD16dim (11/µl vs. 15/µl; *p* = 0.003). For all of these subsets no significant differences could be seen for the advanced tumor group compared to the healthy controls. For all other lymphocyte subsets significant differences, if present, were comparable to those found for the entire patient cohort compared to healthy controls.

Microsatellite stability status did not lead to significant differences between MSI-H or MSS patients in univariate analysis. However this factor was included in the multivariate analysis due to its known impact on the immunogenicity [12, 13, 22].

Univariate analysis of tumor sidedness revealed significantly higher values for total CD8 + cells, memory, effector memory, late and terminal effector CD8 + cells as well as memory, Th17 and HLA-DR CD4 + cells in patients with left sided tumors. Table S2. Chi-square test revealed no significant difference in the distribution of MSI-H and MSS patients between left- and right-sided colorectal carcinomas (*p* = 0.055), although there was a strong trend toward localization in the right hemlicolon for MSI-H patients (10 on the right side vs. 1 on the left side).

Regarding differences according to gender in the whole study population, significant changes were found for naïve CD4 + cells (*p* = 0.026), memory CD8 + cells (*p* = 0.022), CD56bright CD16dim NK cells (*p* = 0.034) and for the CD4/CD8 ratio (*p* = 0.000) in univariate analysis with higher values in women for naïve CD4 cells, CD56bright CD16dim NK cells and CD4/CD8 ratio and higher values for memory CD8 + cells in men.

**Fig. 2:**
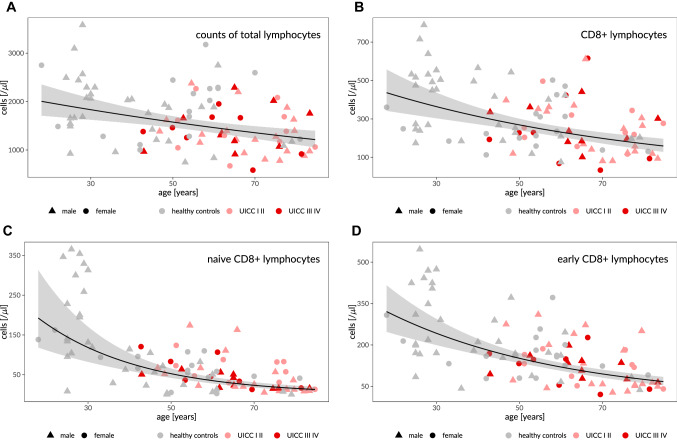
Age-dependent distribution of cell counts and median decrease over lifetime. Black line = smoothed means of log-transformed data, 95% confidence interval in gray. **A** total lymphocytes, **B** CD8 + lymphocytes, **C** naïve CD8 + lymphocytes, **D** early CD8 + lymphocytes in the entire cohort

Univariate analysis of age-dependent changes revealed significant decreases of total lymphocyte count, CD8 + , CD4 + T cells, B cells and NK cells and various of their subsets. An age-dependent increase could be seen for IL2R + CD8 cells, EMRA and HLA-DR CD4 + cells. Percentual alterations of lymphocyte counts per ten years are displayed in Table 3. Most lymphocyte subsets showed a logarithmic decline which is exemplary presented for some subsets in Fig. 2.

**Table 3:** Univariate analysis of age-dependent lymphocyte changes per ten years for the entire cohort (*n* = 97). Cell counts are given as median value/µl (interquartile range)

	Median Cell count/µl (interquartile range)	Relative Cell count deviation per 10 years	*p* value
*Total lymphocytes*	1546.4 (1176.0–2055.7)	− 7.2	**0.000**
CD3 + cells	1047.5 (756.8–1497.3)	− 6.9%	**0.003**
CD8 + cells	252.3 (182.3–423.3)	− 13.3%	**0.000**
Naive	56.1 (20.0–107.4)	− 33.4%	**0.000**
Memory	96.3 (51.2–143.9)	− 12.4%	**0.009**
CM	25.8 (13.6–52.7)	− 10.6%	0.069
EM	79.2 (48.5–135.0)	− 7.0%	0.110
EMRA	50.6 (18.2–115.6)	2.4%	0.714
early	159.5 (79.1–216.3)	− 20.9%	**0.000**
Intermediate	15.0 (8.2–30.3)	− 10.4%	**0.028**
Late	54.1 (23.5–124.5)	− 3.6%	0.553
Exhausted	69.5 (41.8–113.8)	− 7.9%	**0.039**
Terminal effector	29.0 (11.6–97.6)	0.09%	0.990
HLADR +	43.2 (22.0–92.4)	11.2%	0.101
CD69 +	19.8 (11.4–53.6)	− 25.6%	**0.000**
Regulatory	0.3 (0.2–0.6)	− 4.8%	0.593
Il2R +	0.6 (0.3–1.5)	17.0%	**0.035**
CD4 + cells	624.1 (454.7–898.8)	− 8.4%	**0.003**
Naive	226.8 (130.0–372.6)	− 14.8	**0.000**
Memory	340.1 (245.4–466.6)	− 8.4%	**0.006**
CM	220.2 (143.1–290.4)	− 10.9%	**0.001**
EM	123.9 (87.9–200.8)	− 1.3%	0.724
EMRA	3.8 (1.4–24.6)	22.3%	**0.039**
HLADR +	47.2 (32.4–63.0)	8.2%	**0.018**
CD69 +	12.6 (7.6–18.9)	3.3%	0.535
Th1	21.7 (12.4–48.1)	− 1.1%	0.838
Th2	44.8 (31.0–62.5)	4.2%	0.261
Th17	50.7 (34.2–75.1)	− 3.3%	0.387
Regulatory	35.6 (24.0–51.7)	− 6.8%	0.033
Il2R +	15.1 (8.4–21.7)	− 4.7%	0.225
CD3 + CD56 + cells	44.2 (19.0–83.2)	3.8%	0.548
*NK cells*	182.8 (107.8–264.8)	− 6.7%	**0.038**
CD56 + CD16 +	152.9 (90.1–238.1)	− 6.8%	**0.008**
CD56dim CD16bright	13.1 (8.0–19.7)	− 5.1%	0.156
CD56bright CD16dim	12.2 (9.3–17.2)	− 6.6%	0.064
*B cells*	162.2 (100.8–234.0)	− 16.7%	**0.000**
Naive	99.1 (60.1–158.9)	− 19.4%	**0.000**
Memory non-class switch	7.2 (3.7–13.2)	− 14.8%	**0.003**
Memory class switch	19.1 (9.8–31.2)	− 12.0%	**0.009**
Transitional	4.5 (1.6–10.2)	− 38.4%	**0.000**

Bold values indicate lymphocyte subsets with significant differences (*p* < 0.005)

## Multivariate analysis

**Fig. 3:**
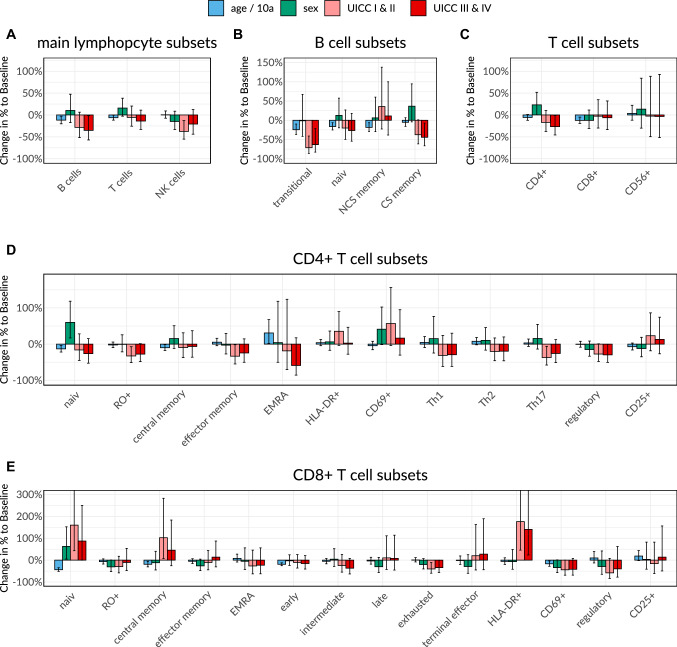
Impact of age, gender and colon carcinoma on lymphocyte subsets as calculated in multivariate analysis. **A** Main lymphocyte subsets, **B** B cell subsets, **C** T cell subsets, **D** CD4 + T cell subsets, **E** CD8 + T cell subsets

In multivariate analysis including age, gender and colon carcinoma in UICC stage I/II or III/IV, significant differences of B cells, CD4 cells and NK cells as seen in the univariate analysis could be attributed to the presence of carcinoma independently of age or gender. Regarding subgroups of these lymphocytes, patients with colon carcinoma had lower transitory B cells and class switched B cells, memory, effector memory, regulatory and Th17 CD4 cells as well as CD56 + CD16 + NK. Total number of CD8 + cells were not altered in colon carcinoma patients in contrast to various cytotoxic T cell subsets as naïve, central memory, exhausted and HLADR + CD8 + cells. Interestingly, most significant differences were found in the lower tumor stage group with exception of CD4 + cells, regulatory CD4 + cells, total B cells and class switched B cells. For some subpopulations differences could be observed additionally in the higher tumor stage group UICC III/ UICC IV. Naïve, central memory and HLADR + CD8 + cells showed increases in tumor patients in contrast to all other subgroups with significant changes where a decline of absolute lymphocyte counts could be seen. Figure 3, Table S3. The multiplicative changes (coefficient B) in cell counts described in Table S2 and percentual changes in Fig. 3 are to be interpreted in the context of a baseline-patient which is male and healthy.

Multivariate analysis revealed an important role of age for lymphocyte evolution. A significant age-dependent decline could be seen for total CD3 + and CD8 + cells, naïve, central memory, early and CD69 + cytotoxic T cells as well as naïve and central memory T helper cells. Total B cells and their subgroups of naïve, non-class-switched memory and transitional B cells showed a significant age-dependent decline as well. For EMRA CD4 + cells and CD4/CD8 ratio an increase with age could be seen. Figure 3, Table S3.

Gender had a significant influence on naïve CD8 + cells, total CD4 + cells, naïve CD4 + cells, CD4/CD8 ratio and CD56 + CD16- NK cells with higher values in women in multivariate analysis. Figure 3, Table S3.

In a second step an additional multivariate analysis was performed in patients only including age, gender, tumor stage, tumor sidedness and MSI. MSI status had no influence on the number of lymphocytes and their subsets with the exception of effector memory CD8 + cells which showed higher values in patients with microsatellite instability. Regarding sidedness of the tumor, localization in the left hemicolon led to higher values of total cytotoxic T cells, effector memory and late CD8 + cells as well as HLADR + T helper cells. Table S4.

## Discussion

Colorectal carcinoma patients presented with significantly lower values of absolute circulating B cell, T cell (CD4 + and CD8 +) and NK cell counts including various subsets compared to healthy volunteers in univariate analysis. Regarding results of previously published studies with similar questions, our results are comparable to a small analysis of Spacek et al. who revealed a decline in absolute counts of CD4 + , CD8 + and NK cells in 22 colon carcinoma patient compared to 22 healthy volunteers [7]. Two studies dealing with proportional values of lymphocyte subsets led to few or no significant differences between colon carcinoma patients and controls [5, 6]. Additionally, these two studies showed significant age differences between patients and healthy controls that were not addressed in a multivariate analysis.

To exclude a bias due to age or gender caused by a higher age in the patient group in particular, we added a multivariate analysis of our study population which revealed a significant decline attributed to colon cancer for a high number of circulating lymphocyte subsets. For some subsets however, age or gender turned out being confounders. Figure 3, Table S3. The influence of aging on different lymphocyte subsets, leading to a decline of naïve T cells and increase in memory and activated T cells is well described in the literature [8, 10]. In our study population in multivariate analysis the previously seen age-dependent decline of naïve T cells and other subgroups could be reproduced.

The gender dependent differences with higher values of total T helper cells and CD4/CD8 ratio in women are consistent with prior analyses [8, 23]. Additionally, higher numbers of naïve CD4 + and CD8 + cells were seen in women which could be explained by an earlier immunosenescence in men [8].

Regarding sidedness of the tumor, localization in the left hemicolon led to significant higher values of several T cell subgroups compared to right-sided tumors in univariate and multivariate analysis whereas no significantly elevated lymphocyte values could be detected for right-sided tumors. The fact that left- and right-sided tumors differ according to their embryological origin, genetics and clinical presentation is well-known since several years [14, 19]. Right-sided colon carcinomas show a distinct pattern of genetic mutations with a higher rate of microsatellite instability and higher mutational burden as well as a higher expression of PD-L1 [14, 15, 20]. Whether theses factors result in an increased, respectively, reduced immunogenicity remains unclear as microsatellite instability is associated with a higher immunogenicity [24] whereas PD-L1 leads to an impaired immune response [25]. Presumed that peripheral blood lymphocytes correlate directly with tumor-infiltrating lymphocytes, our data from the multivariate analysis suggest that right-sided tumors are less immunogenic.

As mentioned above, microsatellite instability is associated with a higher rate of genetic mutations in tumors and with higher immunogenicity [24, 26]. This is displayed by a higher number of tumor-infiltrating CD8 + cells in microsatellite instable tumors [27, 28]. Whether there is a correlation also with peripheral blood cellular immune response has not been investigated so far. In our study univariate analysis revealed no significant differences in lymphocyte counts between MSI-H and MSS patients. Due to the clear indications in the literature that MSI status plays a role in tumor immunogenicity it was still included in multivariate analysis, where significant higher values of effector memory CD8 cells in patients with MSI-H could be detected indicating a more active cellular immune system. For the majority of other CD4 + and CD8 + T cells at least a nonsignificant trend toward higher values in MSI-H patients could be seen in the multivariate analysis. Additionally, the distribution between MSI-H and MSS patients in left- and right-sided colon carcinomas was not significantly different in Chi-square test (*p* = 0.055) as it would have been expected according to the mentioned publications. Again a strong trend toward localization in the right hemicolon for MSI-H patients was observed. Taking together these findings we assume that the relatively low number of 11 MSI-H patients was not sufficient to reach statistical significance, which is a first limitation of our study.

Our study has some further limitations. First of all, our analysis of peripheral blood lymphocytes cannot answer so far whether the detected alterations of peripheral blood lymphocyte subsets in colon carcinoma patients compared to healthy controls correlate with the local immune response to the tumor consisting of tumor-infiltrating lymphocytes and local lymph node activation. A small study on 18 colon carcinoma patients by Hagland et al. revealed a positive correlation between peripheral blood CD8 + , CD4 + and NK cells and tumor-infiltrating CD3 + and CD8 + cells [29]. These results suggest that a higher tumor immunogenicity leads to relatively increased numbers of peripheral blood lymphocytes.

This leads to a second limitation of our study. The question whether tumor patients develop reduced counts of circulating lymphocytes as a consequence of cancer or whether a preexisting lymphopenia favors the development of cancer cannot be answered. An impaired immune response due to immunosuppressive factors in carcinoma patients has been postulated by several studies revealing decreased absolute lymphocyte cell counts in patients with hematological or solid tumors including colorectal carcinoma [30, 31] as well as altered lymphocyte subsets [2–4, 7]. Tumor cells are considered to induce an immunosuppressive, tumor-favorable microenvironment [32, 33]. Whether these alterations are also associated with immunosuppression-associated infections or viral reactivation remains mostly unclear as most studies are considering only patients with solid tumors under chemotherapy and no treatment naïve patients. In a small collective of 17 patients with solid tumors and cytomegalovirus reactivation, however, at least two of these patients had never received chemotherapy at the time of virus reactivation [34]. A preexisting underlying immunosuppression in our colorectal carcinoma cohort could be mostly excluded as patients with chronic infections, inherent or acquired immunodeficiency or immunosuppressive treatment due to autoimmune disorders were excluded.

A third limitation is that the cohort of patients is rather small. Especially those with stage IV tumors were underrepresented as this subgroup consisted only of patients who were scheduled for surgery on the assumption of a metastatic free situation. However, significant differences could be detected despite the small number of patients.

In summary our study revealed significant alterations of circulating lymphocyte subsets that can be attributed to the presence of colon carcinoma despite an important influence of age and gender that should always be considered when regarding lymphocyte subsets. Additionally, certain tumor characteristics like tumor sidedness seem to make a difference in the individual immune response. Whether these lymphocyte alterations in peripheral blood play a role in predicting the prognosis of colon cancer patients as it was reported for tumor-infiltrating lymphocytes [35, 36] and whether the alterations are cause or consequence of the tumor disease will need further investigations.

### Supplementary Information

Below is the link to the electronic supplementary material.

Supplementary file1 (PDF 569 KB)

## Data Availability

The data generated in this study are available upon request from the corresponding author.

## Abbreviations

CRC: Colorectal carcinoma
CTLA-4: Cytotoxic T lymphocyte antigen 4
ECD: Phycoerythrin Texas red
ERMA: Effector memory RA +
FITC: Fluoreszeinisothiocyanat
HLA: Human leucocyte antigen
MMRd: Mismatch repair deficiency
MSI: Microsatellite instability status
MSI-H: Microsatellite instability high
MSS: Microsatellite stable
NK cells: Natural killer cells
NSCLC: Non-small-cell lung cancer
PC5: Phycoerythrin-cyanin
PE: Phycoerythrin
TMB: Tumor mutational burden

## Code availability

No applicable.
