# Supplementary material for: Alterations of circulating lymphocyte subsets in patients with colorectal carcinoma
Source: Cancer Immunol Immunother. 2021 Dec 20;71(8):1937–47. doi: 10.1007/s00262-021-03127-8 (PMC9293872; doi:10.1007/s00262-021-03127-8)
Supplement: Supplementary file 1 — Supplementary file1 (PDF 569 KB) [file 262_2021_3127_MOESM1_ESM.pdf]

Table S1: Fluorochrome-antibody conjugates

Cell population	Antigen	Fluorochrome	Clone	Isotype	Beckman Coulter/Biolegend item number
B	IgD	FITC	IA6-2	IgG2a	B30652
B	CD24	PE	ALB9	IgG1	IM1428U
B, exhausted T	CD27	ECD	1A4CD27	IgG1	B26603
B	CD38	PC5	LS198-4-3	IgG1	A07780
B	CD19	PC7	J3-119	IgG1 kappa	IM3628
Memory T	CD62L	FITC	DREG56	IgG1	IM1231U
Memory T	CD45 R0	PE	UCHL1	IgG2a	A07787
Memory T, Treg	CD45RA	ECD	2H4LDH11LDB9	IgG1	IM2711U
Memory T, activated T, Treg, Th subsets	CD4	PC5	13B8.2	IgG1	A07752
Memory T, Treg, exhausted T, activated T	CD8	PC7	SFCI21Thy2D3	IgG1	737661
Exhausted CD8-T	CD57	FITC	NC1	IgM	B49188
Exhausted CD8-T	CD279	PE	PD1.3	IgG2b	B30634
Exhausted CD8-T	CD28	PC5	CD28.2	IgG1	6607108
Activated T	HLA-DR	FITC	Immu-357	IgG1	IM1638U
Activated T	CD69	PE	TP1.55.3	IgG2b	IM1943U
Treg	CD25	FITC	B1.49.9	IgG2a	IM0478U
Treg	FoxP3	PE	Ab259D	IgG1 kappa	B46031
T/NK	CD56	PE	N901/NK-1	IgG1	A07788
T/NK	CD16	ECD	3G8	IgG1	A33098
T/NK	CD19	PC5	J3-119	IgG1	A07771
T/NK	CD3	PC7	UCHT1	IgG1	737657
Th subsets	CD183 (CXCR3)	AF488	G025H7	IgG1	B68144
	CD194 (CCR4)	PE	L291H4	IgG1	359412
	CD195 (CCR5)	PE/Dazzle 594	J418F1	IgG2b	359126
	CD196 (CCR6)	PC7	B-R35	IgG2a	B68132

FITC fluorescein isothiocyanate, *PE* phycoerythrin, *ECD* phycoerythrin-Texas red, *PC-5* phycoerythrin cyanin, *PC7* phycoerythrin cyanin7, *AF488* Alexa-Fluor 488

Table S2 - Lymphocyte subsets in patients according to tumor sidedness

	**Colon carcinoma patients with right sided tumors** (interquartile range) (n=32)	**Colon carcinoma patients with left sided tumors** Median cell count (interquartile range) (n=15)	**P value univariate analysis**
**Total lymphocytes**	1288 (993 – 1651)	1351 (1070 – 1952)	0.438
**CD3+ cells**	833 (713 – 1121)	952 (714 – 1484)	0.171
**CD8+ cells**	212 (118 – 316)	255 (181 – 441)	**0.040**
Naive	41 (16 – 66)	33 (16 – 73)	0.964
Memory	67 (37 – 107)	97 (55 – 137)	**0.040**
CM	23 (12 – 46)	22 (15 – 55)	0.553
EM	59 (37 – 68)	96 (74 – 216)	**0.004**
EMRA	30 (16 – 99)	83 (25 – 165)	0.077
Early	90 (56 – 168)	135 (58 – 198)	0.424
Intermediate	10 (6 – 24)	15 (8 – 27)	0.240
Late	39 (14 – 92)	94 (63 – 261)	**0.005**
Exhausted	51 (24 – 88)	64 (42 – 89)	0.326
Terminal effector	17 (8 –72)	89 (43 – 207)	**0.003**
Regulatory	0 (0 – 1)	0 (0 – 0)	0.583
IL2	1 (0 – 2)	1 (0 – 1)	0.288
HLA-DR	58 (30 – 109	85 (59 – 117)	0.065
CD69	13 (9 – 25	15 (6 – 46)	0.802
**CD4+ cells**	499 (331 – 626)	590 (439 – 880)	0.096
Naive	145 (77 – 279)	231 (121– 352)	0.157
Memory	272 (187 – 365)	335 (274 – 435)	**0.047**
CM	177 (109 – 234)	208 (162 – 250)	0.137
EM	116 (75– 151)	137 (83 – 189)	0.329
EMRA	6 (1 – 21)	4 (2 – 15)	0.981
Th1	15 (10 – 38)	31 (17 – 42)	0.193
Th2	40 (21 – 58)	57 (39 – 70)	0.131
Th17	39 (29 – 59)	58 (41 – 82)	**0.036**
Regulatory	32 (23 – 40)	25 (13 – 54)	0.819
Il2R+	15 (8 – 27)	15 (8 – 23)	0.749
HLA-DR	47 (35 – 63)	56 (52 –67)	**0.045**
CD69	13 (7 – 17)	13 (11 – 22)	0.349
CD3+ CD56+ cells	37 (15 – 67)	44 (21 – 195)	0.254
**NK cells**	171 (87 – 236)	142 (104 – 210)	0.732
CD56+ CD16+	142 (57 – 214)	108 (75 – 168)	0.616
CD56dim CD16bright	9 (5 – 20)	15 (8 – 18)	0.338
CD56bright CD16dim	11 (9 – 15)	10 (7 – 12)	0.118
**B cells**	125 (80 – 172)	111 (59 – 199)	0.819
Naive	73 (51– 113)	74 (27 – 150)	0.842
Non-class-switched Memory	7 (3 – 15)	6 (3 – 11)	0.767
Class switched	17 (8 – 28)	15 (7 – 20)	0.616
Transitory	2 (1 – 3)	3 (1 – 5)	0.294
**CD4/CD8 Ratio**	2.7 (1.7 – 3.9)	2.1 (1.8 – 3.2)	0.268

Table S3 – Multivariate analysis of lymphocyte subsets regarding age, gender and colon carcinoma

	Variables	Coefficients B (95% CI)	p
**Total Lymphocytes**	age (/10 years)	0.9554 (0.9086-1.0045)	0.0739
	gender	1.0595 (0.9133-1.2291)	0.4413
	UICC I/II	0.8296 (0.6773-1.0160)	0.0704
	UICC III/IV	0.8312 (0.6720-1.0281)	0.0874
**Total Tcells (CD3+)**	age (/10 years)	0.9381 (0.8837-0.9958)	**0.0361**
	gender	1.1597 (0.9718-1.3838)	0.0994
	UICC I/II	0.9463 (0.7434-1.2046)	0.6507
	UICC III/IV	0.8624 (0.6697-1.1107)	0.2484
**Cytotoxic Tcells (CD8+)**	age (/10 years)	0.8772 (0.8091-0.9510)	**0.0018**
	gender	0.8770 (0.6905-1.1140)	0.2787
	UICC I/II	0.9731 (0.7020-1.3488)	0.8684
	UICC III/IV	0.9380 (0.6661-1.3210)	0.7113
**CD8+ naive**	age (/10 years)	0.5645 (0.4870-0.6542)	**0.0000**
	gender	1.6271 (1.0496-2.5224)	**0.0299**
	UICC I/II	2.6004 (1.4292-4.7314)	**0.0021**
	UICC III/IV	1.8705 (0.9993-3.5013)	0.0503
**CD8+ memory**	age (/10 years)	0.9328 (0.8210-1.0597)	0.2814
	gender	0.6919 (0.4743-1.0093)	0.0558
	UICC I/II	0.7021 (0.4193-1.1758)	0.1764
	UICC III/IV	0.8956 (0.5216-1.5377)	0.6863
**CD8+ CM**	age (/10 years)	0.8085 (0.6910-0.9459)	**0.0085**
	gender	0.8815 (0.5530-1.4052)	0.5924
	UICC I/II	2.0245 (1.0712-3.8261)	**0.0303**
	UICC III/IV	1.4537 (0.7463-2.8317)	0.2679
**CD8+ EM**	age (/10 years)	0.9477 (0.8433-1.0651)	0.3636
	gender	0.7386 (0.5221-1.0448)	0.0861
	UICC I/II	0.8948 (0.5572-1.4367)	0.6420
	UICC III/IV	1.1393 (0.6938-1.8709)	0.6028
**CD8+ EMRA**	age (/10 years)	1.0788 (0.9126-1.2752)	0.3704
	gender	0.9468 (0.5752-1.5584)	0.8280
	UICC I/II	0.7359 (0.3715-1.4578)	0.3752
	UICC III/IV	0.7667 (0.3767-1.5605)	0.4596
**CD8+ early**	age (/10 years)	0.8095 (0.7442-0.8804)	**0.0000**
	gender	0.9709 (0.7571-1.2450)	0.8140
	UICC I/II	0.8952 (0.6375-1.2571)	0.5188
	UICC III/IV	0.8445 (0.5916-1.2056)	0.3483
**CD8+ intermediate**	age (/10 years)	0.9463 (0.8332-1.0747)	0.3910
	gender	1.0435 (0.7161-1.5206)	0.8228
	UICC I/II	0.7543 (0.4511-1.2613)	0.2789
	UICC III/IV	0.6300 (0.3675-1.0800)	0.0920
**CD8+ late**	age (/10 years)	0.9622 (0.8189-1.1306)	0.6364
	gender	0.6985 (0.4334-1.1257)	0.1387
	UICC I/II	1.1019 (0.5743-2.1142)	0.7680
	UICC III/IV	1.0810 (0.5459-2.1406)	0.8213
**CD8+ exhausted**	age (/10 years)	1.0098 (0.9151-1.1142)	0.8450
	gender	0.8002 (0.5980-1.0709)	0.1322

	UICC I/II	0.5971 (0.4011-0.8888)	**0.0116**
	UICC III/IV	0.6562 (0.4324-0.9958)	**0.0478**
**CD8+ TE**	age (/10 years)	0.9805 (0.8082-1.1895)	0.8402
	gender	0.7055 (0.3983-1.2498)	0.2288
	UICC I/II	1.2034 (0.5512-2.6273)	0.6387
	UICC III/IV	1.2751 (0.5624-2.8911)	0.5569
**CD8+ CD69+**	age (/10 years)	0.8336 (0.7208-0.9640)	**0.0147**
	gender	0.6584 (0.4282-1.0125)	0.0568
	UICC I/II	0.5580 (0.3101-1.0041)	0.0516
	UICC III/IV	0.5809 (0.3137-1.0755)	0.0832
**CD8+ HLADR**	age (/10 years)	0.9437 (0.8056-1.1056)	0.4693
	gender	0.9315 (0.5832-1.4880)	0.7643
	UICC I/II	2.7608 (1.4565-5.2330)	**0.0022**
	UICC III/IV	2.4073 (1.2313-4.7066)	**0.0108**
**CD8+ Il2R+**	age (/10 years)	1.1865 (0.9794-1.4373)	0.0799
	gender	1.0340 (0.5862-1.8239)	0.9071
	UICC I/II	0.8395 (0.3868-1.8221)	0.6550
	UICC III/IV	1.1364 (0.5043-2.5607)	0.7553
**CD8+ FoxP3+**	age (/10 years)	1.1005 (0.8705-1.3913)	0.4192
	gender	0.7087 (0.3541-1.4185)	0.3270
	UICC I/II	0.4188 (0.1624-1.0801)	0.0713
	UICC III/IV	0.6019 (0.2229-1.6252)	0.3127
**T helper cells (CD4+)**	age (/10 years)	0.9436 (0.8793-1.0126)	0.1055
	gender	1.2324 (1.0001-1.5186)	**0.0499**
	UICC I/II	0.8306 (0.6245-1.1047)	0.1994
	UICC III/IV	0.7343 (0.5446-0.9902)	**0.0431**
**CD4+ naive**	age (/10 years)	0.8674 (0.7809-0.9635)	**0.0085**
	gender	1.5997 (1.1707-2.1858)	**0.0036**
	UICC I/II	0.8376 (0.5469-1.2827)	0.4109
	UICC III/IV	0.7372 (0.4717-1.1520)	0.1782
**CD4+ memory**	age (/10 years)	0.9768 (0.9020-1.0578)	0.5602
	gender	0.9937 (0.7850-1.2578)	0.9576
	UICC I/II	0.6725 (0.4874-0.9278)	**0.0162**
	UICC III/IV	0.7226 (0.5157-1.0126)	0.0590
**CD4+ CM**	age (/10 years)	0.8995 (0.8217-0.9846)	**0.0222**
	gender	1.1543 (0.8828-1.5092)	0.2905
	UICC I/II	0.9060 (0.6253-1.3128)	0.5983
	UICC III/IV	0.9341 (0.6369-1.3701)	0.7246
**CD4+ EM**	age (/10 years)	1.0530 (0.9562-1.1596)	0.2901
	gender	0.9724 (0.7288-1.2973)	0.8473
	UICC I/II	0.6655 (0.4508-0.9824)	**0.0406**
	UICC III/IV	0.7543 (0.4968-1.1452)	0.1831
**CD4+ EMRA**	age (/10 years)	1.3075 (1.0200-1.6760)	**0.0346**
	gender	1.0415 (0.4971-2.1820)	0.9132
	UICC I/II	0.8115 (0.2942-2.2382)	0.6835
	UICC III/IV	0.4089 (0.1424-1.1740)	0.0956
**CD4+CD69+**	age (/10 years)	0.9547 (0.8454-1.0782)	0.4513
	gender	1.4116 (0.9848-2.0233)	0.0603
	UICC I/II	1.5689 (0.9597-2.5650)	0.0721
	UICC III/IV	1.1643 (0.6954-1.9494)	0.5591

**CD4+HLADR**	age (/10 years)	1.0384 (0.9549-1.1293)	0.3739
	gender	1.0616 (0.8283-1.3605)	0.6335
	UICC I/II	1.3538 (0.9648-1.8997)	0.0791
	UICC III/IV	1.0269 (0.7199-1.4648)	0.8823
**THC 1**	age (/10 years)	1.0460 (0.9056-1.2083)	0.5368
	gender	1.1521 (0.7519-1.7652)	0.5116
	UICC I/II	0.6887 (0.3820-1.2416)	0.2119
	UICC III/IV	0.7069 (0.3839-1.3019)	0.2622
**THC 2**	age (/10 years)	1.0779 (0.9810-1.1843)	0.1170
	gender	1.1031 (0.8348-1.4575)	0.4861
	UICC I/II	0.7967 (0.5422-1.1706)	0.2437
	UICC III/IV	0.8038 (0.5395-1.1976)	0.2793
**THC 17**	age (/10 years)	1.0321 (0.9353-1.1388)	0.5256
	gender	1.1551 (0.8633-1.5455)	0.3280
	UICC I/II	0.6285 (0.4204-0.9398)	**0.0241**
	UICC III/IV	0.7402 (0.4879-1.1229)	0.1550
**CD4+ Il2R+**	age (/10 years)	0.9283 (0.8380-1.0283)	0.1519
	gender	0.8775 (0.6483-1.1878)	0.3936
	UICC I/II	1.2327 (0.8154-1.8637)	0.3174
	UICC III/IV	1.1301 (0.7327-1.7432)	0.5763
**CD4+ FoxP3+**	age (/10 years)	0.9926 (0.9144-1.0776)	0.8586
	gender	0.8496 (0.6663-1.0833)	0.1861
	UICC I/II	0.7244 (0.5199-1.0095)	0.0568
	UICC III/IV	0.7020 (0.4957-0.9941)	**0.0463**
**CD3+ CD56+**	age (/10 years)	1.0380 (0.8817-1.2220)	0.6511
	gender	1.1354 (0.7005-1.8404)	0.6027
	UICC I/II	0.9746 (0.5040-1.8846)	0.9384
	UICC III/IV	0.9660 (0.4838-1.9285)	0.9210
**Total B cells (CD19+)**	age (/10 years)	0.8821 (0.7997-0.9728)	**0.0126**
	gender	1.1042 (0.8264-1.4756)	0.4986
	UICC I/II	0.7166 (0.4823-1.0646)	0.0979
	UICC III/IV	0.6494 (0.4288-0.9834)	**0.0416**
**Naïve B cells**	age (/10 years)	0.8368 (0.7472-0.9372)	**0.0024**
	gender	1.1258 (0.8044-1.5755)	0.4856
	UICC I/II	0.7983 (0.5025-1.2682)	0.3362
	UICC III/IV	0.7333 (0.4559-1.1796)	0.1982
**Non-class switched memory B cells**	age (/10 years)	0.8142 (0.7086-0.9354)	**0.0041**
	gender	1.0611 (0.7036-1.6002)	0.7749
	UICC I/II	1.3573 (0.7746-2.3784)	0.2823
	UICC III/IV	1.1106 (0.6168-1.9998)	0.7239
**Class switched memory B cells**	age (/10 years)	0.9466 (0.8399-1.0669)	0.3645
	gender	1.3656 (0.9585-1.9455)	0.0837
	UICC I/II	0.6246 (0.3852-1.0127)	0.0562
	UICC III/IV	0.5577 (0.3360-0.9256)	**0.0244**
**Transitional B cells**	age (/10 years)	0.7538 (0.6306-0.9012)	**0.0022**
	gender	0.9858 (0.5812-1.6721)	0.9573
	UICC I/II	0.2864 (0.1392-0.5892)	**0.0009**
	UICC III/IV	0.3673 (0.1724-0.7825)	**0.0100**
**Natural killer cells**	age (/10 years)	1.0058 (0.9264-1.0920)	0.8897
	gender	0.8522 (0.6681-1.0871)	0.1953
	UICC I/II	0.6263 (0.4492-0.8732)	

	UICC III/IV	0.7935 (0.5600-1.1243)	**0.0063**<br>0.1906
**CD56+ CD16+**	age (/10 years)	1.0283 (0.9371-1.1284)	0.5526
	gender	0.7854 (0.5967-1.0339)	0.0843
	UICC I/II	0.5589 (0.3840-0.8134)	**0.0027**
	UICC III/IV	0.6955 (0.4692-1.0308)	0.0700
**CD56bright CD16dim**	age (/10 years)	0.9538 (0.8958-1.0155)	0.1372
	gender	1.2473 (1.0360-1.5016)	**0.0201**
	UICC I/II	0.8271 (0.6420-1.0656)	0.1402
	UICC III/IV	0.8771 (0.6725-1.1440)	0.3295
**CD56dim CD16bright**	age (/10 years)	1.0058 (0.9076-1.1147)	0.9107
	gender	0.7646 (0.5641-1.0363)	0.0829
	UICC I/II	0.6849 (0.4522-1.0374)	0.0735
	UICC III/IV	0.8744 (0.5658-1.3513)	0.5417
**CD4/CD8 ratio**	age (/10 years)	1.0754 (1.0038-1.1521)	**0.0389**
	gender	1.4031 (1.1443-1.7204)	**0.0014**
	UICC I/II	0.8543 (0.6467-1.1286)	0.2644
	UICC III/IV	0.7838 (0.5854-1.0495)	0.1008

Table S4 – Multivariate analysis of lymphocyte subsets regarding MSI, sidedness and colon carcinoma

	Variables	Coefficients B (95% CI)	p
**Total Lymphocytes**	age (/10 years)	0.9414 (0.8503-1.0423)	0.2375
	gender (female)	1.0060 (0.8000-1.2651)	0.9579
	UICC III/IV	0.9694 (0.7581-1.2396)	0.7993
	Sidedness (left)	1.0704 (0.8374-1.3683)	0.5780
	MSI status (stable)	1.0810 (0.8101-1.4426)	0.5878
**Total Tcells (CD3+)**	age (/10 years)	0.9384 (0.8308-1.0600)	0.2977
	gender (female)	1.1597 (0.8045-1.3922)	0.6781
	UICC III/IV	0.8989 (0.6698-1.2064)	0.4678
	Sidedness (left)	0.8624 (0.9084-1.6347)	0.1811
	MSI status (stable)	0.9928 (0.7030-1.4022)	0.9666
**Cytotoxic Tcells (CD8+)**	age (/10 years)	0.8968 (0.7498-1.0727)	0.2258
	gender (female)	0.9257 (0.6187-1.3850)	0.7003
	UICC III/IV	0.9359 (0.6074-1.4420)	0.7580
	Sidedness (left)	1.5965 (1.0368-2.4582)	**0.0344**
	MSI status (stable)	0.7857 (0.4731-1.3048)	0.3419
**CD8+ naive**	age (/10 years)	0.6076 (0.4968-0.7430)	**0.0000**
	gender (female)	1.4957 (0.9510-2.3523)	0.0798
	UICC III/IV	0.7256 (0.4464-1.1795)	0.1893
	Sidedness (left)	0.9761 (0.6009-1.5854)	0.9200
	MSI status (stable)	1.2412 (0.7019-2.1949)	0.4476
**CD8+ memory**	age (/10 years)	0.9848 (0.7522-1.2894)	0.9090
	gender (female)	0.5059 (0.2759-0.9277)	**0.0286**
	UICC III/IV	1.4965 (0.7807-2.8683)	0.2174
	Sidedness (left)	1.7976 (0.9388-3.4419)	0.0755
	MSI status (stable)	0.5305 (0.2473-1.1381)	0.1009
**CD8+ CM**	age (/10 years)	0.9314 (0.7008-1.2378)	0.6157
	gender (female)	0.6767 (0.3568-1.2833)	0.2243
	UICC III/IV	0.7213 (0.3630-1.4332)	0.3415
	Sidedness (left)	1.1664 (0.5877-2.3153)	0.6520
	MSI status (stable)	1.1704 (0.5229-2.61959	0.6949
**CD8+ EM**	age (/10 years)	1.0182 (0.8281-1.2518)	0.8610
	gender (female)	0.6698 (0.4208-1.0661)	0.0890
	UICC III/IV	1.4698 (0.8926-2.4204)	0.1263
	Sidedness (left)	2.1366 (1.2986-3.5156)	**0.0038**
	MSI status (stable)	0.4746 (0.2644-0.8521)	0.0139
**CD8+ EMRA**	age (/10 years)	0.9515 (0.6314-0.9201)	0.7720
	gender (female)	1.1220 (0.5156-2.4412)	0.7659
	UICC III/IV	0.8593 (0.3683-2.0049)	0.7189
	Sidedness (left)	2.0924 (0.8954-4.8895)	0.0862
	MSI status (stable)	0.8066 (0.3026-2.1498)	0.6595
**CD8+ early**	age (/10 years)	0.7622 (0.7442-0.8804)	**0.0059**
	gender (female)	0.9058 (0.5929-1.3836)	0.6390
	UICC III/IV	0.8726 (0.5539-1.3748)	0.5476
	Sidedness (left)	1.1060 (0.7025-1.7413)	0.6556
	MSI status (stable)	1.1106 (0.6515-1.8931)	0.6929

**CD8+ intermediate**	age (/10 years)	1.0437 (0.7835-1.3903)	0.7645
	gender (female)	0.9222 (0.4837-1.7582)	0.8008
	UICC III/IV	0.9351 (0.4679-1.8688)	0.8455
	Sidedness (left)	1.5683 (0.7856-3.1307)	0.1954
	MSI status (stable)	0.6500 (0.2885-1.4646)	0.2898
**CD8+ late**	age (/10 years)	1.0536 (0.7644-1.4523)	0.7435
	gender (female)	1.1601 (0.5634-2.3886)	0.6795
	UICC III/IV	0.9207 (0.4242-1.9982)	0.8302
	Sidedness (left)	2.9727 (1.3714-6.4438)	**0.0070**
	MSI status (stable)	0.6706 (0.2702-1.6646)	0.3792
**CD8+ exhausted**	age (/10 years)	1.0129 (0.8206-1.2504)	0.9023
	gender (female)	0.7850 (0.4887-1.2610)	0.3078
	UICC III/IV	1.2072 (0.7260-2.0073)	0.4582
	Sidedness (left)	1.1995 (0.7219-1.9929)	0.4728
	MSI status (stable)	0.8168 (0.4498-1.4833)	0.4965
**CD8+ TE**	age (/10 years)	1.0472 (0.7202-1.5227)	0.8044
	gender (female)	1.1200 (0.4823-2.6007)	0.7869
	UICC III/IV	0.9444 (0.3824-2.3320)	0.8987
	Sidedness (left)	4.1628 (1.6882-10.2645)	**0.0028**
	MSI status (stable)	0.5749 (0.1991-1.6603)	0.2973
**CD8+ CD69+**	age (/10 years)	0.8062 (0.6144-1.0578)	0.1167
	gender (female)	0.8807 (0.4779-1.6228)	0.6762
	UICC III/IV	1.1732 (0.6089-2.2604)	0.6248
	Sidedness (left)	0.9955 (0.5172-1.9161)	0.9890
	MSI status (stable)	0.9072 (0.4203-1.9584)	0.7993
**CD8+ HLADR**	age (/10 years)	1.1098 (0.8524-1.4450)	0.4290
	gender (female)	1.0059 (0.5554-1.8216)	0.9842
	UICC III/IV	1.0632 (0.5622-2.0105)	0.8468
	Sidedness (left)	2.2533 (1.1927-4.2569)	**0.0137**
	MSI status (stable)	0.4767 (0.2257-1.0067)	0.0520
**CD8+ Il2R+**	age (/10 years)	1.2672 (0.8883-1.8076)	0.1851
	gender (female)	1.1697 (0.5260-2.6015)	0.6935
	UICC III/IV	1.3546 (0.5746-3.1935)	0.4781
	Sidedness (left)	0.6594 (0.2801-1.5525)	0.3311
	MSI status (stable)	0.9920 (0.3627-2.7134)	0.9872
**CD8+ FoxP3+**	age (/10 years)	1.3011 (0.8064-2.0995)	0.2724
	gender (female)	1.0433 (0.3555-3.0620)	0.9369
	UICC III/IV	1.7968 (0.5660-5.7045)	0.3110
	Sidedness (left)	0.8223 (0.2595-2.6060)	0.7333
	MSI status (stable)	0.6644 (0.1713-2.5769)	0.5451
**T helper cells (CD4+)**	age (/10 years)	0.9118 (0.7833-1.0615)	0.2724
	gender (female)	1.1138 (0.7912-1.5680)	0.9369
	UICC III/IV	0.8420 (0.5834-1.2153)	0.3110
	Sidedness (left)	1.3862 (0.9610-1.9995)	0.7333
	MSI status (stable)	0.9931 (0.6457-1.5274)	0.5451
**CD4+ naive**	age (/10 years)	0.8640 (0.6716-1.1115)	0.2475
	gender (female)	1.4557 (0.8258-2.5659)	0.1879
	UICC III/IV	0.8420 (0.4583-1.5469)	0.5704
	Sidedness (left)	1.6344 (0.8905-2.9996)	0.1097
	MSI status (stable)	0.9848 (0.4824-2.0102)	0.9655

**CD4+ memory**	age (/10 years)	0.9500 (0.8037-1.1229)	0.5384
	gender (female)	0.7861 (0.5395-1.1453)	0.2032
	UICC III/IV	1.0891 (0.7273-1.6309)	0.6711
	Sidedness (left)	1.4299 (0.9555-2.1398)	0.0805
	MSI status (stable)	0.8159 (0.5080-1.3103)	0.3900
**CD4+ CM**	age (/10 years)	0.9619 (0.8286-1.1167)	0.6013
	gender (female)	0.9236 (0.6594-1.2937)	0.6357
	UICC III/IV	1.0497 (0.7346-1.5000)	0.7845
	Sidedness (left)	1.2848 (0.8990-1.8361)	0.1634
	MSI status (stable)	1.0597 (0.6917-1.6234)	0.7847
**CD4+ EM**	age (/10 years)	0.9647 (0.7870-1.1825)	0.7223
	gender (female)	0.8444 (0.5323-1.3396)	0.4621
	UICC III/IV	1.2265 (0.7463-2.0158)	0.4101
	Sidedness (left)	1.1579 (0.7004-1.9145)	0.5579
	MSI status (stable)	0.6279 (0.3564-1.1062)	0.1043
**CD4+ EMRA**	age (/10 years)	0.9578 (0.5952-1.5411)	0.8552
	gender (female)	1.5371 (0.5263-4.4889)	0.4216
	UICC III/IV	0.3838 (0.1193-1.2340)	0.1051
	Sidedness (left)	1.2696 (0.3940-4.0911)	0.6818
	MSI status (stable)	0.9875 (0.2557-3.8146)	0.9851
**CD4+CD69+**	age (/10 years)	0.9056 (0.7438-1.1025)	0.3141
	gender (female)	1.3605 (0.8738-2.1185)	0.1674
	UICC III/IV	0.7307 (0.4543-1.1751)	0.1892
	Sidedness (left)	1.4130 (0.8792-2.2707)	0.1484
	MSI status (stable)	0.8155 (0.4670-1.4241)	0.4636
**CD4+HLADR**	age (/10 years)	1.0737 (0.9234-1.2486)	0.3458
	gender (female)	1.0263 (0.7309-1.4411)	0.8778
	UICC III/IV	0.7419 (0.5154-1.0680)	0.1054
	Sidedness (left)	1.5496 (1.0772-2.2292)	**0.0195**
	MSI status (stable)	0.8391 (0.5473-1.2865	0.4111
**THC 1**	age (/10 years)	0.9737 (0.7107-1.3341)	0.8649
	gender (female)	1.2380 (0.6044-2.5358)	0.5499
	UICC III/IV	0.9451 (0.4419-2.0214)	0.8812
	Sidedness (left)	1.5492 (0.7245-3.3124)	0.2507
	MSI status (stable)	0.7784 (0.3114-1.9458)	0.5829
**THC 2**	age (/10 years)	0.9739 (0.7906-1.1998)	0.7990
	gender (female)	0.9466 (0.5888-1.5219)	0.8161
	UICC III/IV	0.8628 (0.5215-1.4275)	0.5562
	Sidedness (left)	1.3279 (0.8028-2.1966)	0.2609
	MSI status (stable)	1.4066 (0.7668-2.5804)	0.2619
**THC 17**	age (/10 years)	0.9266 (0.7408-1.1590)	0.4942
	gender (female)	1.1329 (0.6806-1.8857)	0.6228
	UICC III/IV	0.9407 (0.5481-1.6148)	0.8200
	Sidedness (left)	1.4598 (0.8506-2.5053)	0.1642
	MSI status (stable)	1.6395 (0.8549-3.1440)	0.1324
**CD4+ Il2R+**	age (/10 years)	1.1919 (0.9512-1.4934)	0.1234
	gender (female)	0.9076 (0.5464-1.5077)	0.7013
	UICC III/IV	1.0756 (0.6240-1.8541)	0.7880
	Sidedness (left)	0.8848 (0.5137-1.5239)	0.6511
	MSI status (stable)	0.9904 (0.5228-1.8761)	0.9757

**CD4+ FoxP3+**	age (/10 years)	0.9389 (0.7607-1.1589)	0.5482
	gender (female)	0.8338 (0.5192-1.3390)	0.4420
	UICC III/IV	0.9423 (0.5669-1.5665)	0.8142
	Sidedness (left)	0.9692 (0.5835-1.6099)	0.9014
	MSI status (stable)	1.0844 (0.5974-1.9687)	0.7846
**CD3+ CD56+**	age (/10 years)	0.8972 (0.6464-1.2453)	0.5069
	gender (female)	0.9553 (0.4568-1.9980)	0.9008
	UICC III/IV	0.9181 (0.4160-2.0264)	0.8282
	Sidedness (left)	1.4822 (0.6724-3.2674)	0.3199
	MSI status (stable)	0.8692 (0.3433-2.2006)	0.7617
**Total B cells (CD19+)**	age (/10 years)	0.7130 (0.5696-0.8925)	**0.0042**
	gender (female)	0.8915 (0.5378-1.4777)	0.6480
	UICC III/IV	0.8170 (0.4751-1.4051)	0.4552
	Sidedness (left)	0.9412 (0.5477-1.6173)	0.8220
	MSI status (stable)	1.1092 (0.5871-2.0956)	0.7434
**Naïve B cells**	age (/10 years)	0.6781 (0.5171-0.8894)	**0.0062**
	gender (female)	0.8176 (0.4439-1.5060)	0.5084
	UICC III/IV	0.8721 (0.4562-1.6669)	0.6710
	Sidedness (left)	0.9408 (0.4939-1.7921)	0.8488
	MSI status (stable)	1.0251 (0.4766-2.2050)	0.9480
**Non-class switched memory B cells**	age (/10 years)	0.6394 (0.4566-0.8955)	**0.0106**
	gender (female)	0.7851 (0.3680-1.6753)	0.5221
	UICC III/IV	0.8550 (0.3791-1.9280)	0.6986
	Sidedness (left)	0.8095 (0.3595-1.8231)	0.6013
	MSI status (stable)	0.7641 (0.2943-1.9838)	0.5714
**Class switched memory B cells**	age (/10 years)	0.7252 (0.5764-0.9126)	**0.0074**
	gender (female)	1.3394 (0.7986-2.2463)	0.2597
	UICC III/IV	0.7316 (0.4201-1.2742)	0.2613
	Sidedness (left)	0.9677 (0.5561-1.6838)	0.9051
	MSI status (stable)	1.2185 (0.6355-2.3362)	0.5425
**Transitional B cells**	age (/10 years)	0.5680 (0.3969-0.8128)	**0.0028**
	gender (female)	0.5341 (0.2384-1.1966)	0.1238
	UICC III/IV	1.0360 (0.4360-2.4616)	0.9346
	Sidedness (left)	1.0086 (0.4250-2.3932)	0.9842
	MSI status (stable)	1.6438 (0.5955-4.5378)	0.3280
**Natural killer cells**	age (/10 years)	1.1433 (0.9565-1.3665)	0.1369
	gender (female)	0.8343 (0.5585-1.2463)	0.3665
	UICC III/IV	1.3358 (0.8684-2.0548)	0.1815
	Sidedness (left)	0.8023 (0.5219-1.2333)	0.3063
	MSI status (stable)	1.2580 (0.7590-2.0851)	0.3636
**CD56+ CD16+**	age (/10 years)	1.1520 (0.9443-1.4054)	0.1577
	gender (female)	0.7951 (0.5083-1.2436)	0.3059
	UICC III/IV	1.3233 (0.8189-2.1385)	0.2447
	Sidedness (left)	0.7645 (0.4735-1.2345)	0.2638
	MSI status (stable)	1.3748 (0.7829-2.4144)	0.2596
**CD56bright CD16dim**	age (/10 years)	1.0638 (0.9445-1.1981)	0.2991
	gender (female)	1.3113 (1.0035-1.7136)	**0.0472**
	UICC III/IV	1.1533 (0.8655-1.5367)	0.3210
	Sidedness (left)	0.8383 (0.6294-1.1165)	0.2204
	MSI status (stable)	1.0355 (0.7394-1.4502)	0.8350

**CD56dim CD16bright**	age (/10 years)	1.2356 (0.9985-1.5291)	0.0516
	gender (female)	0.8017 (0.4963-1.2950)	0.3566
	UICC III/IV	1.5735 (0.9405-2.6323)	0.0825
	Sidedness (left)	1.2124 (0.7253-2.0267)	0.4525
	MSI status (stable)	0.8274 (0.4524-1.5133)	0.5291
**CD4/CD8 ratio**	age (/10 years)	1.0166 (0.8659-1.1935)	0.8365
	gender (female)	1.2019 (0.8377-1.7245)	0.3090
	UICC III/IV	0.9003 (0.6112-1.3263)	0.5864
	Sidedness (left)	0.8683 (0.5898-1.2783)	0.4644
	MSI status (stable)	1.2624 (0.8013-1.9887)	0.3059

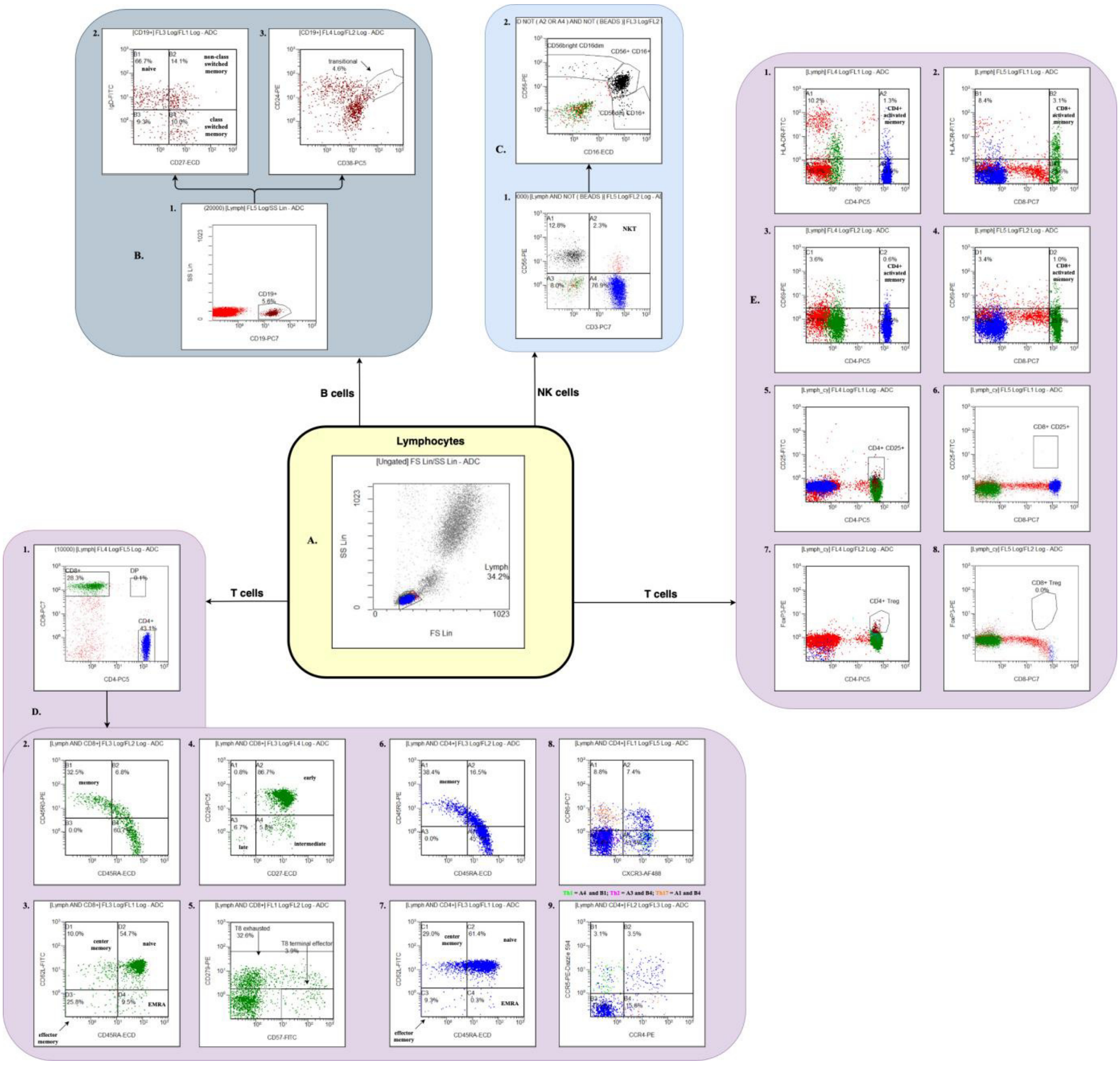

Supplemental Figure 1: **Gating Strategy.** (**A**.) Lymphocytes were identified by using forward scatter (FC) and side scatter (SC). (**B**.) Additional CD19 positivity was used to define B cells (Plot 1), which were further subdivided into naïve (IgD+), class switched memory (CD27+), non-class switched memory (IgD+ CD27+, Plot 2) and transitional B cells (CD24+ CD38+, Plot 3). (**C**.) NKT cells (CD3+ CD56+) were primarily identified (Plot 1). CD3- cells were classified into 3 different subgroups of NK cells (CD56+ CD16+, CD56bright CD56 dim, CD56dim CD16bright, Plot 2).

(D.) T cells were identified by CD4 or CD8 positivity. Both CD4+ as well as CD8+ cells were subdivided into memory (CD45RA- CD45RO+) (Plots 1+6), naïve (CD62L+ CD45RA+), central memory (CD62L+ CD45RA-), effector memory (CD62L- CD45RA-) and effector memory RA+ ("EMRA") (CD62L- CD45RA+) cells (Plots 3+7). To analyze CD8+ cell activity, cells were subdivided into early (CD27+ CD28+), intermediate (CD27+ CD28-), late (CD27- CD28-) (Plot 4), or exhausted (CD279+) and terminal effector (CD279- CD57+) cells (Plot 5). CD4+ T helper cells were classified into Th1 (CXCR3+ CCR4- CCR5+ CCR6-), Th2 (CXCR3- CCR4+ CCR5- CCR6-) and Th17 (CXCR3- CCR4+ CCR5- CCR6+) cells (Plots 8+9). **(E.)** CD4+ and CD8+ cells were also subdivided into activated memory cells (HLA-DR+ or CD69+) (Plots 1-4) and regulatory cells (CD25+ or FoxP3+) (Plots 5-8).
